# Increasing value and reducing waste in data extraction for systematic reviews: tracking data in data extraction forms

**DOI:** 10.1186/s13643-017-0546-z

**Published:** 2017-08-04

**Authors:** Farhad Shokraneh, Clive E. Adams

**Affiliations:** 1 Cochrane Schizophrenia Group, The Institute of Mental Health, A Partnership Between The University of Nottingham and Nottinghamshire Healthcare NHS Trust, Nottingham, UK; 2 Research Center for Modeling in Health, Institute for Futures Studies in Health, Kerman University of Medical Sciences, Kerman, Iran

**Keywords:** Data extraction, Systematic reviews, Traceable data, Data location, Portable Document Format (PDF), Increasing value, Reducing waste

## Abstract

Data extraction is one of the most time-consuming tasks in performing a systematic review. Extraction is often onto some sort of form. Sharing completed forms can be used to check quality and accuracy of extraction or for re-cycling data to other researchers for updating. However, validating each piece of extracted data is time-consuming and linking to source problematic.

In this methodology paper, we summarize three methods for reporting the location of data in original full-text reports, comparing their advantages and disadvantages.

**Electronic supplementary material:**

The online version of this article (doi:10.1186/s13643-017-0546-z) contains supplementary material, which is available to authorized users.

## Main text

### Background

One of the time-consuming tasks in conducting a systematic review is data extraction and should be done by at least two researchers to reduce error [1, 2]. Traditionally, the research team uses a form unto which they enter extracted data. These forms then become the dataset and can be made open access for reuse––a practice that has been encouraged for some time [3].

Although sharing data extracted from reports is an attractive option, research has identified that––understandably––extraction errors are common (20/34 Cochrane systematic reviews [4]). Verifying laboriously extracted data, however, necessitates re-locating the text from which the data were extracted in the original report. Such re-locating of each tiny data-point in full texts may require the same amount of time that the original review team already spent and is duplication of effort.

Tracking extracted data to the original source is valuable for checking quality [4] and to ensure ease of reuse [3]. In this paper, we highlight three techniques for making the extracted data traceable to source.

### First method: simple annotation

**Fig. 1:**
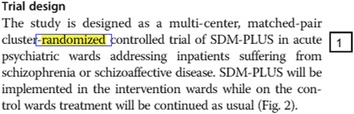
Examples of three tracking methods in PDF. *The number in the text box* is the result of using *simple annotation* method. *The highlighted and linked box* is the result of *Cartesian coordinate system*. Descriptive addressing method does not require in PDF file, and based on the data extraction form, we could find the data in PG2TrialDesignL2 (page 2, trial design, line 2)

This method is similar to citing/referencing system in science/technology literature. We highlight the related data and then annotate a number to it on the original full text and then refer to this number in data extraction form (Table 1, Fig. 1).

**Table 1:** Example of using *simple annotation* method in data extraction form

Design	Location in PDF
Randomized

Although this has the advantage of simplicity, sharing completed data extraction forms will not be helpful without also sharing the same annotated source document. Annotations are valid only in the company of the specific source file that has been used by the research team. Copyright may not allow sharing the PDF files.

### Second method: descriptive addressing

In this method, the “address” of each data point is extracted. For example, in the case of PDF files, the structure includes pages, paragraphs, lines, tables, figures, boxes, and headlines (Table 2, Fig. 1).

**Table 2:** Example of using *descriptive addressing* method in data extraction form

Design	Location in PDF
Randomized	PG2TrialDesignL2

To provide an example of how this may be shared, as a part of a funded project [5], we extracted the data from all randomized trials relevant to treatment of a disorder of movement and made them available [6]. This has the advantage of being the only PDF-independent method. If the data extraction forms are available then sharing the PDFs is not required. The readers could access the PDF file from the journal’s website and locate the data by following the address.

### Third method: Cartesian coordinate system

Every single pixel in a particular PDF file has a unique address. Each word can be identified within a rectangle as a two-dimensional object (Table 3, Fig. 1).

**Table 3:**
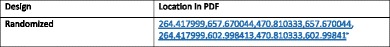
Example of using ‘*Cartesian coordinate system*’ method in data extraction form

* This is not a real link but mimicking a link to show the possibility of linking from the data extraction form to the location of the data within the PDF

This system is similar to––but not the same as––Global Positioning System (GPS) for geographical location. Whereas GPS has one source document (the Earth) and therefore co-ordinates and universally applicable, reviewers may be using different PDFs of the same document. One may be a photocopy of the report published within the journal. Another may be the downloaded PDF of the same report. Co-ordinates on one PDF will not tally with another. This method is in its infancy, but with increasing interest from computer sciences [7, 8] and increasing quality and uniformity of PDF, this method is promising for the automation of data tracking. Co-ordinates make it possible to link from the data extraction form to the location of data-point inside the PDF.

### Comparing methods

The first two methods are usable by anyone; the last is computerized and has the potential to be fully automated, but it is not yet available for systematic reviewers. Extraction may be an ongoing process, and update is important. The data systematic reviewers extracted from a study 10 years ago are of ongoing value but rarely contained the detail necessitated by modern standards that is now routine. Ease of appending existing data extraction forms is important (Table 4).

**Table 4:** Comparing the three methods of tracking extracted data

Methods	Advantages	Disadvantages
Simple annotation	• Available • Easy	• Full texts must be available • Ties user to original highlighted PDF • Difficult to update • Requires PDF editor
Descriptive addressing	• Available • Applicable to any PDF of same report • Update is possible • No editing required in PDF	• Full texts must be available • Less easy than simple annotation • Uniformity of location definition could be problematic
Cartesian coordinates	• Possibility of hyperlinking from data to report • Possibility of automating data quality check • Ease of update	• Full texts must be available • Piloting––unavailable to wide use

### Conclusions

All three methods require access to the original document, so efforts to make research results open-access are of ongoing importance. We think the future is the human-machine interaction and is likely to be driven by Cartesian co-ordinates relating to uniform PDF reports. The human interface of such a system would be a package to upload or relate to the highest quality uniformly available PDF to highlight text from which the data are extracted to the form, carrying their co-ordinates with them via hyperlink. Until that is widely available, we suggest the second method (descriptive addressing) to locate original source data (see Additional file 1).

## Additional file

Additional file 1: Data extraction form for systematic review of randomized clinical trials. (DOCX 14 kb)

## Abbreviations

PDF: Portable Document Format
