# Supplementary material for: Increasing value and reducing waste in data extraction for systematic reviews: tracking data in data extraction forms
Source: Syst Rev. 2017 Aug 4;6:153. doi: 10.1186/s13643-017-0546-z (PMC5544999; doi:10.1186/s13643-017-0546-z)
Supplement: Additional file 1: — Data extraction form for systematic review of randomized clinical trials. (DOCX 14 kb) [file 13643_2017_546_MOESM1_ESM.docx]

Additional file 1: A data extraction form based on real data using *Descriptive addressing* method

| **Study Name** | **Jahanian 2014** | | | |  |
| --- | --- | --- | --- | --- | --- |
| Reference | [Ref. ID 19855] Jahanian AA, Rezaei O, Fadai F, Yaraghchi A. The Effectiveness of Rivastigmine in Reducing Tardive Dyskinesia Symptoms in Patients with Schizophrenia. Iranian Journal of Psychiatry and Clinical Psychology 2014; 20(1): 29-34. | | | |  |
|  | | | | | |
| **Characteristics** |  | | | | **Location in PDF*** |
| Methods | Allocation: "randomly assigned" no details reported. | | | | 19855PG30C1P3L7 |
|  | Blindness: "double blind" no details reported. | | | | 19855PG30C1P3L3 |
|  | Design: not reported. | | | |  |
|  | Duration: "eight weeks". | | | | 19855PG31C1P2L4 |
|  | Setting: "Razi Psychiatric Center, Tehran, Iran". | | | | 19855PG30C1P3L5 |
| Participants | Diagnosis: Patients with schizophrenia and tardive dyskinesia (TD) based on DSM-IV-TR diagnosed by psychiatrist. | | | | 19855PG30C1P3L12-13 |
|  | N=40. | | | | 19855PG30C1P3L5 |
|  | Age: range 18-65 years. | | | | 19855PG30C1P3L17 |
|  | Sex: not reported. | | | |  |
| Interventions | 1. Rivastigmine: dose: 1.5 mg twice daily. N=20. | | | | 19855PG30C1P3L7-8 |
|  | 2. Placebo: no details reported. N=20. | | | | 19855PG30C1P3L10 |
| Outcomes | TD symptoms: no improvement (AIMS). | | | | 19855PG31C1P2L5 |
| Notes | Sponsorship source: "no financial support". | | | | 19855PG33C2P3L1-2 |
| **Risk of Bias** | | | | | |
| Bias | Support Statement from Report | | | |  |
| Random sequence generation | "Randomly". No details. | | | | 19855PG30C1P3L7 |
| Allocation concealment | Not reported. | | | |  |
| Blinding of participants and personnel | "Double blind". No details. | | | | 19855PG30C1P3L3 |
| Blinding of outcome assessment | "Double blind". No details. | | | | 19855PG30C1P3L3 |
| Incomplete outcome data | Not reported. | | | |  |
| Selective reporting | Outcomes have been reported based on the registered protocol IRCT2012092910964N1. | | | | 19855PG30C1P3L1 |
| Other biases | None known. | | | |  |
| **Outcome** | | | | | |
|  | Rivastigmine | | Placebo | |  |
|  | Mean | SD | Mean | SD |  |
| AIMS after Intervention | 12.5 | 7.0 | 10.3 | 3.1 | 19855PG32T2 |

*In this example, the first five digits refer to the file name, PG to pages, C to column, P to page, L to line, and T to Table.
